# The importance of apnea in patients undergoing angiography of thoracic vessels – a protocol for acute pulmonary thromboembolism research

**DOI:** 10.1590/S1679-45082017AI3884

**Published:** 2017

**Authors:** Kátia Ayumi Takeda, Walther Yoshiharu Ishikawa, Camila dos Santos Silva, Fábio Augusto, Elaine Ferreira da Silva, Adriano Tachibana, Marcelo Buarque de Gusmão Funari

**Affiliations:** 1 Hospital Israelita Albert Einstein, São Paulo, SP, Brazil.

This was 37-year-old woman, 10 days after surgery, who underwent breast prosthesis replacement, abdominoplasty and liposuction. Upon admission, she was hemodynamically stable, febrile and reported constant pain on left hemithorax region, but without dyspnea.

**Figure 1:**
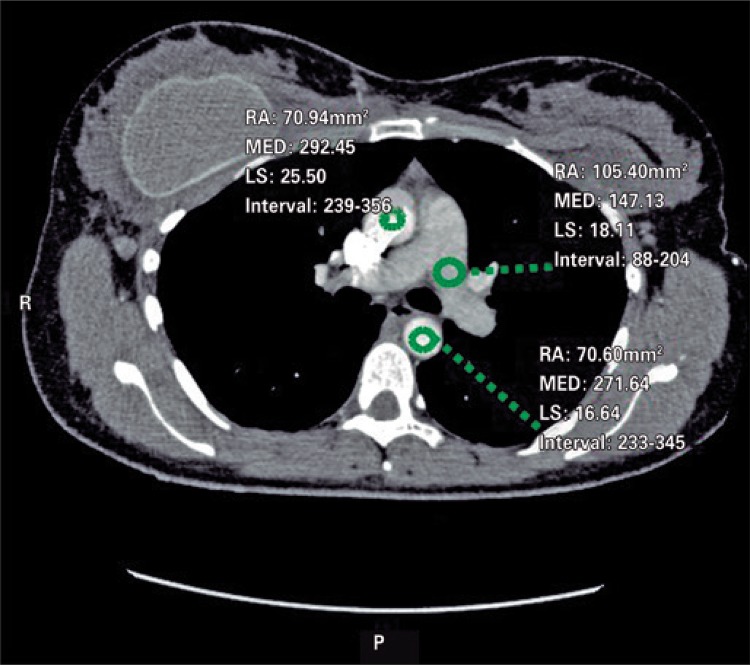
Contrast agent density in pulmonary artery of 147.13, contrast agent density in ascending aorta of 292.45, and contrast agent density in descending aorta of 271.64

**Figure 2:**
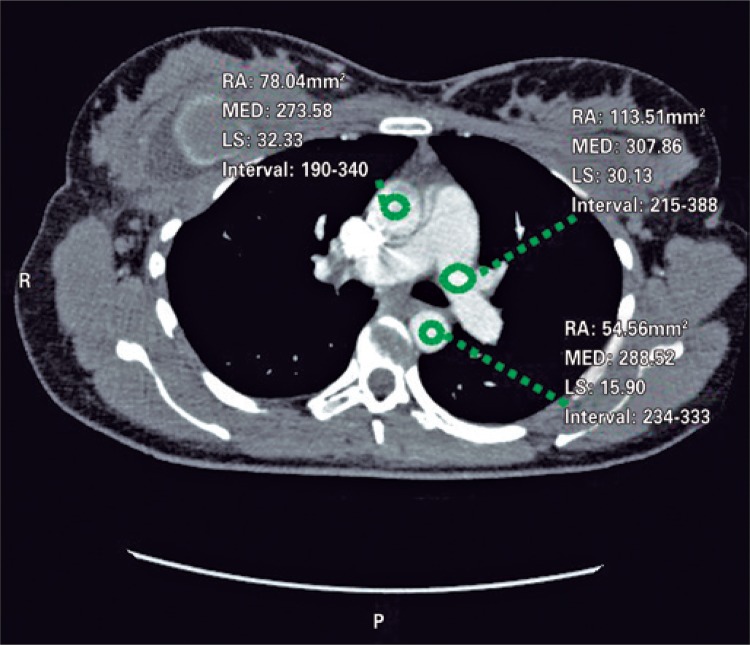
Contrast agent density in pulmonary artery of 307.86, contrast agent in ascending aorta of 273.58 and contrast agent of descending aorta of 288.52

**Figure 3:**
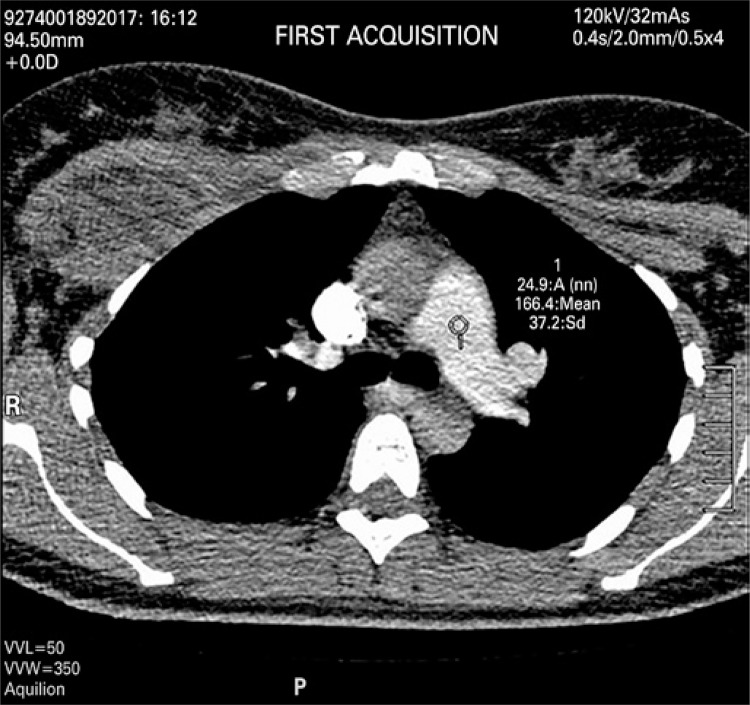
Contrast agent density in pulmonary artery of 166.4 for acquisition of exam sequence

**Figure 4:**
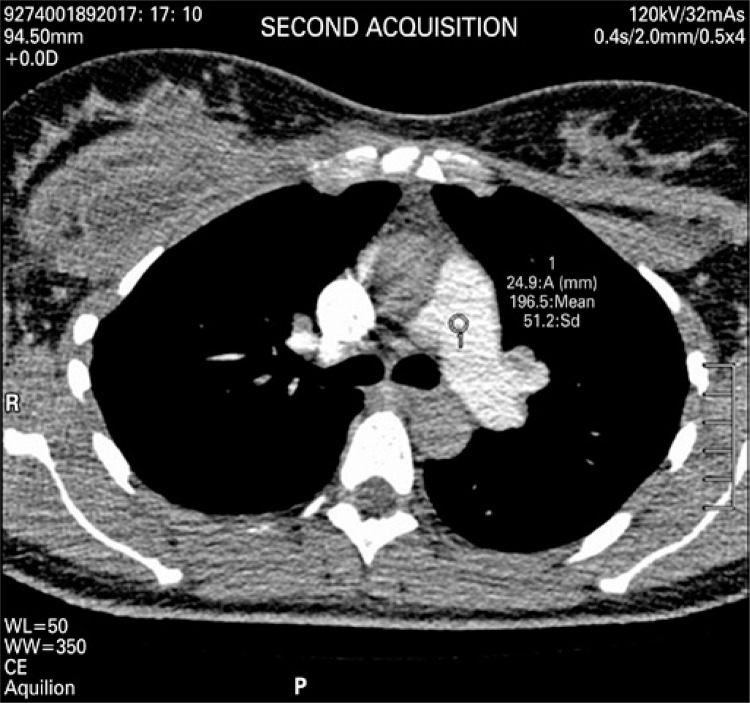
Contrast agent density in pulmonary artery of 196.5 for acquisition of second sequence

An angio-CT of thoracic vessels was carried out. During intravenous injection of contrast agent, the patient was anxious and started crying during the exam. We believe that, a Valsalva maneuver was performed on inspiration apnea requested during the exam therefore causing an intrathoracic pressure that resulted only in a thoracic aortic contrast (Figure 1). A new contrast agent was intravenously injected after apnea orientation, and the Valsalva maneuver was not requested, which resulted in precisely diagnostic images (Figure 2). Two acquisitions were carried out using manual trigger when peak of pulmonary artery contrast occurred (Figure 3 and 4).

The diagnosis of acute pulmonary thromboembolism (PTE) is based on clinical probability, use of D-dimer dosage and imaging assessment – including the angio-CT because it is a rapid, non-invasive procedure with high sensibility and specificity (83% and 96%, respectively).^1^ This method enables to evaluate all area of mediastinum and pulmonary parenchyma, and, the use of intravenous iodine contrast agent in its maximal peak enables to evaluate the pulmonary artery and its distal branches and thoracic aorta.

Studies show that negative angio-CT exam, even in case of good quality images, is enough to exclude PTE.^2,3^ An important factor that affects negatively the quality of exam is the transitory interruption of contrast agent, which was first described by Gosselin et al., as a physiologic artifact,^4^ which also entails low contrast of pulmonary artery and its segments. This vascular phenomena must occur when patient undergo a short time deep inhaling before image acquisitions, which result in an increase of blood venous return to the inferior vena cava, or reduction in contrast agent flow reduction by the superior vena cava. The non-opacified blood in left atrium dilutes the contrast agent of the superior vena cava,^5^ therefore causing low attenuation of pulmonary artery and difficult to diagnose PTE.
